# Individualized cognitive-behavioral therapy improves anxiety, depression, sleep quality, hopelessness, and disease severity in fibromyalgia syndrome: a single-blind randomized controlled trial

**DOI:** 10.1007/s00296-026-06146-9

**Published:** 2026-05-26

**Authors:** Burhan Fatih Kocyigit, Gülşah Yaşa Öztürk, Mehmet Macit Sönmez, Saltanat K. Yerkebayeva, Meirgul I. Assylbek

**Affiliations:** 1 Department of Physical Medicine and Rehabilitation, University of Health Sciences, Adana City Research and Training Hospital, Adana, Türkiye; 2 Adana City Research and Training Hospital, Adana, Türkiye; 3 Department of Neurology, Psychiatry, Rehabilitation and Neurosurgery, South Kazakhstan Medical Academy, Shymkent, Kazakhstan; 4 Department of Social Health Insurance and Public Health, South Kazakhstan Medical Academy, Shymkent, Kazakhstan; 5 Medical Center “Mediker”, Shymkent, Kazakhstan

**Keywords:** Fibromyalgia, Cognitive behavioral therapy, Psychotherapy, Depression, Anxiety, Hope, Sleep

## Abstract

The current study evaluates the effects of individualized cognitive-behavioral therapy (CBT), administered alongside routine treatment, on anxiety, depression, sleep quality, hopelessness, and disease severity in patients with fibromyalgia syndrome (FMS). This is a randomized, controlled, single-blind clinical trial was conducted in a tertiary physical therapy and rehabilitation clinic. Seventy patients who met the 2016 American College of Rheumatology diagnostic criteria were randomized in a 1:1 ratio into an intervention group (*n* = 35) and a control group (*n* = 35). The intervention group received a 10-week individualized CBT program administered by a psychologist in addition to routine treatment, while the control group received only routine treatment. Outcomes were assessed using the hospital anxiety and depression Scale (HADS), comprising the anxiety subscale (HADS-A) and the depression subscale (HADS-D), Jenkins sleep scale (JSS), fibromyalgia impact questionnaire (FIQ), and Beck hopelessness scale (BHS) before and after the intervention. A total of 57 patients completed the study, with 23 in the intervention group and 34 in the control group. No significant differences were found between the groups in baseline demographic characteristics and scale scores (*p* > 0.05). The intervention group demonstrated statistically significant improvements in all scales following treatment: the HADS-A median score decreased from 13.00 to 10.00 (*p* < 0.001), the FIQ median score decreased from 71.80 to 51.52 (*p* < 0.001), and the BHS median score decreased from 7.00 to 4.00 (*p* < 0.001). In contrast, the control group showed no significant changes on any scale (*p* > 0.05). Individualized CBT, added to routine treatment, addresses anxiety, depression, sleep quality, disease impact, and other issues in FMS patients. Integrating individualized CBT protocols into clinical practice for fibromyalgia management should be encouraged.

**Supplementary Information:**

The online version contains supplementary material available at 10.1007/s00296-026-06146-9.

## Introduction

Fibromyalgia syndrome (FMS) is a persistent musculoskeletal disorder marked by unrelenting pain and heightened sensitivity throughout the body. Those affected often grapple with exhaustion, mental fog, a host of physical symptoms, and emotional challenges like depression and anxiety [1, 2]. The reach of FMS stretches far beyond the person diagnosed, disrupting work life and placing a heavy demand on healthcare systems. Annual healthcare visits for patients with FMS are approximately twice those of healthy individuals, and total healthcare costs for these patients are nearly three times higher than those of a random sample [3].

The clinical presentation of FMS extends beyond physical symptoms, as its psychosocial dimension significantly influences disease severity and treatment outcomes. A meta-analysis of depression and anxiety prevalence among FMS patients indicated that approximately 46.6% exhibited symptoms of anxiety, while 50.8% experienced symptoms of depression [4]. Psychiatric comorbidities substantially diminish patients’ quality of life and adversely influence the prognosis of FMS [5]. Sleep disturbances are a primary feature of FMS, with poor sleep quality exacerbating pain perception and sustaining the cycle of fatigue and psychological distress [6]. Furthermore, hopelessness, a cognitive factor closely linked to depression, has been identified as a significant determinant of functional results in chronic pain disorders [7]. Therefore, FMS management requires a holistic approach that addresses both physical and psychosocial aspects.

Non-pharmacological interventions are supported by a growing body of evidence for managing FMS [8] and are emphasized in clinical guidelines. International guidelines prioritize aerobic exercise, cognitive-behavioral therapy (CBT), and patient education to improve pain and related symptoms [9, 10]. The primary objective of CBT in FMS is to improve self-management by facilitating the development of more adaptive beliefs regarding patients’ ability to cope with pain and clinical signs. CBT exerts its effects on psychological symptoms and functional impairment through mechanisms including the restructuring of maladaptive thought patterns, reduction of pain catastrophizing, and regulation of activity [11, 12].

While CBT is effective in reducing pain, negative mood, and disability in FMS [12, 13], few studies have assessed its impact on anxiety, depression, sleep quality, hopelessness, and overall disease impact when delivered individually with a standardized session protocol. This study evaluated the effects of ten weekly individual CBT sessions, administered by a psychologist in addition to routine care, on anxiety, depression, sleep quality, hopelessness, and disease severity in patients with FMS, compared to those receiving routine care alone. The primary outcome was disease severity, while secondary outcomes included anxiety, depression, sleep quality, and hopelessness.

## Methods

This randomized controlled trial was conducted at the Physical Medicine and Rehabilitation clinic of a tertiary healthcare center. All participants received a diagnosis of FMS in the Physical Medicine and Rehabilitation outpatient clinic. The study was conducted between July 15, 2025, and January 15, 2026, and included only patients who met the 2016 American College of Rheumatology (ACR) diagnostic criteria [14]. Individuals with active psychiatric disorders, including psychosis, bipolar disorder, or suicidal ideation, as well as those with substance or alcohol dependence, were excluded from the study. Additional exclusion criteria included a diagnosis of inflammatory rheumatic disease, a history of malignancy, pregnancy or breastfeeding status, receipt of CBT within the past year, illiteracy or inability to understand Turkish, severe cognitive impairment, and physical or social barriers to regular session attendance. Furthermore, participants whose pharmacological treatment was changed during the study period were excluded.

### Randomization procedure

Participants were randomly allocated in a 1:1 ratio to either an intervention group that received routine treatment in addition to cognitive-behavioral therapy or a control group that received only routine treatment. Allocation concealment was ensured using sealed, opaque envelopes. CBT sessions were administered by a single clinical psychologist employed at the hospital. All scale assessments were conducted by one physician in the Physical Medicine and Rehabilitation clinic, who remained blinded to participants’ group assignments. Therefore, the study utilized a single-blind design.

### Data assessment

Demographic data recorded for participants included age, sex, body mass index (BMI), duration of FMS, occupational status, and educational level.

### Hospital anxiety and depression scale

Participants completed the hospital anxiety and depression Scale (HADS), a 14-item survey developed by Zigmond and Snaith. The tool consists of two sections, each with 7 questions rated from 0 to 3, assessing anxiety and depression separately [15]. The scale is divided into two subscales - HADS-Anxiety (HADS-A) and HADS-Depression (HADS-D). Scores indicate whether individuals are within the normal range, at the borderline, or likely experiencing clinically significant symptoms [16].

### Fibromyalgia impact questionnaire

Disease severity and functional status were assessed with the fibromyalgia impact questionnaire (FIQ), developed by Burckhardt et al. [17]. This 10-item instrument measures physical functioning, occupational capacity, overall well-being, pain intensity, fatigue, morning stiffness, and affective symptoms such as anxiety and depression. Composite scores range from 0 to 100, with higher values indicating greater disease burden [18].

### Jenkins sleep scale

The Jenkins sleep scale (JSS) was used to assess sleep quality. Developed by Jenkins et al. [19], the scale comprises four items: difficulty initiating sleep, frequent nocturnal awakenings, difficulty maintaining uninterrupted sleep, and experiencing fatigue despite sufficient sleep duration. Each item is rated from 0 (nearly never) to 5 (22–31 days) based on its frequency over the previous month, yielding a total score of 0 to 20. Higher scores indicate more severe sleep disturbances [20].

### Beck hopelessness scale

The Beck hopelessness scale (BHS) is intended to assess the degree of hopelessness. Developed by Beck et al. [21], the scale comprises 20 items assessing negative expectations about the future. Each item is answered as true or false, yielding a total score of 0 to 20. Higher scores indicate more pessimistic expectations and a greater sense of hopelessness [22].

### Cognitive behavioral therapy

Participants assigned to the intervention group received a CBT program, delivered individually by a psychologist alongside standard pharmacological treatment. The program comprised ten weekly sessions, each lasting approximately 50 to 60 min. The therapy protocol incorporated several CBT techniques that have been shown to be effective in managing FMS. Behavioral activation strategies were implemented to address functional limitations and social withdrawal associated with pain. This approach focused on systematically restructuring daily activities [23].To address kinesiophobia, which is prevalent among individuals with FMS, a gradual exposure technique was implemented. This approach facilitated the controlled and progressive reintroduction of previously avoided physical activities [24].Motivational interviewing techniques were incorporated into the therapy process to address ambivalence toward treatment and enhance self-efficacy [25]. The first two sessions provided psychoeducation on fibromyalgia syndrome (FMS) and introduced the cognitive-behavioral model of chronic pain. Cognitive restructuring strategies helped participants identify and change maladaptive beliefs about pain and catastrophic thinking. Relaxation training, including diaphragmatic breathing and progressive muscle relaxation, was used to reduce physiological arousal. Sleep hygiene education addressed unhelpful sleep habits. The final session focused on relapse prevention and strengthening coping skills. Session materials were adapted to each participant’s symptoms and functional limitations.

### Ethics approval

The study was approved by the local ethics committee (Decision No: 585, Date: July 10, 2025). The study was conducted in accordance with the principles of the Declaration of Helsinki (revised October 2024).

### Statistical analysis

Statistical analyses were conducted using IBM Statistical Package for the Social Sciences (SPSS), version 20. The Shapiro-Wilk test was used to assess the normality of continuous variables. As the data did not meet the assumption of normality, nonparametric tests were employed. Between-group comparisons of baseline characteristics utilized the Mann-Whitney U test for continuous variables and the Chi-square test for categorical variables. Results are reported as median (minimum–maximum) for continuous variables and as number (percentage) for categorical variables. Within-group comparisons of pre- and post-treatment scores were performed using the Wilcoxon signed-rank test. To account for the potential influence of baseline values in intergroup comparisons following the intervention, the difference (delta) between pre- and post-treatment scores was calculated for each participant. The Mann-Whitney U test was then used to compare delta scores between groups. Statistical significance was defined as a p-value less than 0.05. In addition to p-values, 95% confidence intervals for within-group and between-group comparisons were calculated using the Hodges-Lehmann estimator.

## Results

**Fig. 1:**
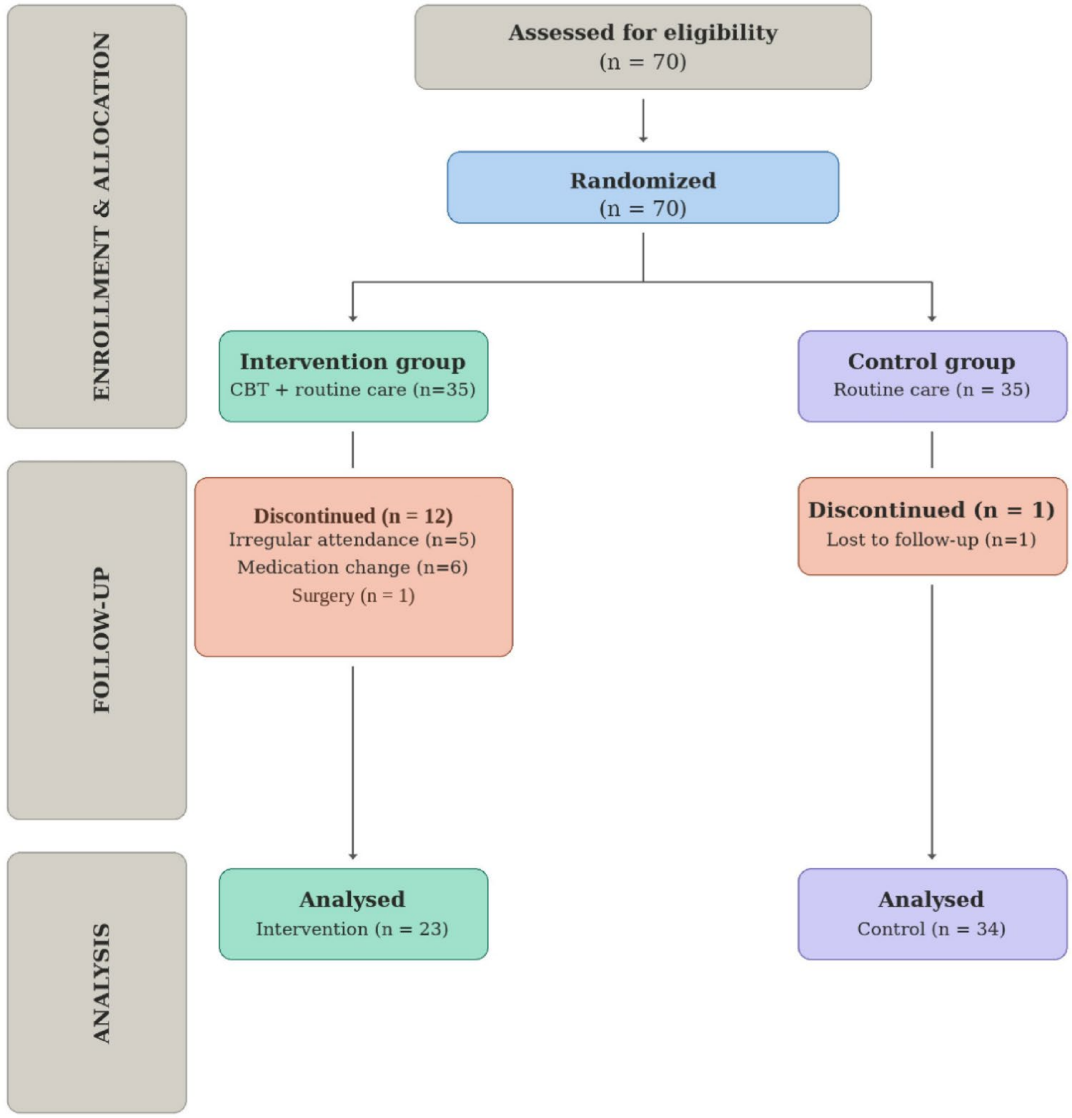
Participant flow through the study

Seventy patients were enrolled in the study, with 35 assigned to each group. One patient in the control group was excluded due to discontinuation of follow-up, resulting in 34 patients in this group. Within the intervention group, five patients were excluded due to irregular attendance at CBT sessions, six due to changes in pharmacological treatment during the study, and one due to undergoing surgery. Consequently, the intervention group comprised 23 patients. Overall, 57 participants completed the study (Fig. 1).

The median age in the intervention group was 54 (29–68) years, compared to 51 (28–66) years in the control group (*p* = 0.397). The median BMI was 27.51 (23.83–37.85) kg/m² in the intervention group and 27.12 (19.38–37.25) kg/m² in the control group (*p* = 0.807). In terms of gender distribution, 95.7% of the intervention group were female (*n* = 22) and 4.3% were male (*n* = 1), while 97.1% of the control group were female (*n* = 33) and 2.9% were male (*n* = 1) (*p* = 0.777). Baseline characteristics, including disease duration, education, and employment status, are summarized in Table 1. No statistically significant difference was found between the groups in terms of all scale scores in the pre-treatment period (*p* > 0.05).

**Table 1:** Baseline characteristics of intervention and control groups

Parameter	Intervention group (*n* = 23)	Control group (*n* = 34)	*p*
Age (years)	54 (29–68)	51 (28–66)	0.397
Body mass index (kg/m²)	27.51 (23.83–37.85)	27.12 (19.38–37.25)	0.807
Disease duration (years)	5 (1–15)	5 (1–30)	0.831
Sex, n (%)			0.777
Female	22 (95.7%)	33 (97.1%)	
Male	1 (4.3%)	1 (2.9%)	
Education level, n (%)			0.186
Primary school	6 (26.1%)	18 (52.9%)	
Middle school	4 (17.4%)	2 (5.9%)	
High school	8 (34.8%)	8 (23.5%)	
University or above	5 (21.7%)	6 (17.6%)	
Employment status, n (%)			0.340
Housewife / Unemployed	21 (91.3%)	28 (82.4%)	
Employed	2 (8.7%)	6 (17.6%)	

Continuous variables are presented as median (minimum–maximum); *n* number, *%* percentage. Mann-Whitney U test was used for continuous variables; Chi-square test for categorical variables

Pre- and post-treatment comparisons were conducted across all scales, including within-group analyses. In the intervention group, the median HADS-A score decreased from 13.00 (5.00–20.00) before treatment to 10.00 (4.00–15.00) after treatment (*p* < 0.001). The median HADS-D score declined from 10.00 (2.00–21.00) to 7.00 (3.00–19.00) (*p* = 0.005). The JSS median score was reduced from 15.00 (9.00–20.00) to 13.00 (3.00–19.00) (*p* < 0.001). The FIQ median score improved from 71.80 (29.20–97.92) to 51.52 (12.30–86.60) (*p* < 0.001). The BHS median score decreased from 7.00 (1.00–20.00) to 4.00 (0.00–14.00) (*p* < 0.001).

Pre- and post-treatment scores were compared for all scales within the control group. The median HADS-A score was 11.50 (4.00–19.00) at baseline and 11.00 (4.00–18.00) at follow-up (*p* = 0.472). The median HADS-D score was 8.00 (2.00–17.00) initially and 8.50 (2.00–18.00) at follow-up (*p* = 0.112). The JSS median score was 12.00 (5.00–20.00) at baseline and 13.00 (6.00–20.00) at follow-up (*p* = 0.908). The median FIQ scores were 61.08 (27.28–97.92) at baseline and 56.95 (28.28–90.02 at follow-up (*p* = 0.943). The BHS median score was 8.00 (1.00–17.00) at baseline and 8.50 (1.00–17.00) at follow-up (*p* = 0.115).

Within-group comparisons of clinical scale scores in the intervention and control groups are presented in Table 2.

**Table 2:** Within-group and between-group delta score comparisons of scale scores in intervention and control groups

Outcome	Intervention group (*n* = 23)				Control group (*n* = 34)				Between-group delta			
	Pre-treatment Median (Min–Max)	Post-treatment Median (Min–Max)	*p*	95% CI (within-group Δ)	Pre-treatment Median (Min–Max)	Post-treatment Median (Min–Max)	*p*	95% CI (within-group Δ)	Intervention Median (Δ)	Control Median (Δ)	*p*	95% CI (between-group Δ)
HADS-Anxiety	13.00 (5.00–20.00)	10.00 (4.00–15.00)	< 0.001	[− 4.00, − 3.50]	11.50 (4.00–19.00)	11.00 (4.00–18.00)	0.472	[0.50, 0.50]	3.00	0.00	< 0.001	[4.00, 4.00]
HADS-Depression	10.00 (2.00–21.00)	7.00 (3.00–19.00)	0.005	[− 1.50, − 1.00]	8.00 (2.00–17.00)	8.50 (2.00–18.00)	0.112	[0.00, 0.50]	2.00	0.00	0.002	[2.00, 2.00]
Jenkins Sleep Scale	15.00 (9.00–20.00)	13.00 (3.00–19.00)	< 0.001	[− 2.00, − 2.00]	12.00 (5.00–20.00)	13.00 (6.00–20.00)	0.908	[0.50, 0.50]	2.00	0.00	< 0.001	[2.00, 3.00]
FIQ	71.80 (29.20–97.92)	51.52 (12.30–86.60)	< 0.001	[− 17.01, − 13.70]	61.08 (27.28–97.92)	56.95 (28.28–90.02)	0.943	[0.77, 1.50]	14.53	0.00	< 0.001	[16.90, 20.20]
BHS	7.00 (1.00–20.00)	4.00 (0.00–14.00)	< 0.001	[− 3.50, − 2.50]	8.00 (1.00–17.00)	8.50 (1.00–17.00)	0.115	[0.00, 0.50]	3.00	0.00	< 0.001	[3.00, 4.00]

Wilcoxon signed-rank test was used for within-group comparisons. Between-group delta scores were compared using the Mann-Whitney U test. 95% confidence intervals were calculated using the Hodges-Lehmann estimator. Δ: change score (pre-treatment minus post-treatment)

*HADS* Hospital Anxiety and Depression Scale, *FIQ* Fibromyalgia Impact Questionnaire, *BHS* Beck Hopelessness Scale, *JSS* Jenkins Sleep Scale

The potential impact of baseline score differences on treatment outcomes was assessed by calculating delta scores for all scales and comparing them between groups. In the intervention group, the median delta scores were 3.00 for HADS-A, 2.00 for HADS-D, 2.00 for JSS, 14.53 for FIQ, and 3.00 for BHS. In contrast, the control group exhibited a median delta score of 0.00 for all scales except FIQ, for which the median delta score was − 0.50. Statistically significant differences favoring the intervention group were observed across all scales when comparing delta scores between groups (HADS-A: *p* < 0.001, HADS-D: *p* = 0.002, JSS: *p* < 0.001, FIQ: *p* < 0.001, BHS: *p* < 0.001) (Table 2).

## Discussion

This randomized controlled trial assessed the impact of ten individually administered CBT sessions on anxiety, depression, sleep quality, hopelessness, and disease severity in patients with FMS. The intervention group demonstrated statistically significant improvements on all measured scales, including HADS-A, HADS-D, JSS, FIQ, and BHS, compared to the control group receiving only routine therapy. The control group exhibited no significant changes on any assessment scale. These results indicate that individualized CBT, when combined with standard pharmacological treatment, offers multidimensional and clinically meaningful benefits for the management of FMS.

In our study, statistically significant decreases in both anxiety and depression scores were observed in the intervention group following treatment. In contrast, the control group showed no significant change in either parameter. These results align with existing literature. A large-sample meta-analysis by Bernardy et al. [12] reported that CBT demonstrated a superior effect on negative mood compared to control conditions, supported by high-quality evidence. A randomized controlled trial investigating individually administered CBT in women with FMS demonstrated significant improvements in depressive symptoms and anxiety levels [26]. The improvement in psychiatric symptoms observed in this study likely resulted from the restructuring of maladaptive thought patterns and the reduction of pain catastrophizing, both of which are central mechanisms of CBT. A neuroimaging-assisted randomized controlled trial by Lee et al. [11] demonstrated that CBT significantly reduced pain-related catastrophizing, and this reduction mediated functional improvement. Furthermore, maladaptive emotion regulation strategies such as rumination and catastrophizing are strong predictors of depression and anxiety in patients with FMS [27]. These findings support the hypothesis that CBT can improve psychological symptoms by targeting these specific cognitive processes.

Sleep disturbance is a core feature of FMS, contributing to a cycle that intensifies pain perception and increases psychological distress [28]. The intervention group demonstrated a significant reduction in JSS scores following treatment. Furthermore, disease severity, as measured by the FIQ, improved in this group. The present findings align with the existing literature, further substantiating the positive effects of CBT on sleep quality and disease severity [24, 29, 30]. The mechanisms underlying the observed improvements in sleep quality and disease severity may involve several interconnected processes. Specifically, CBT reduces pain catastrophizing and hypervigilance, which, in turn, alleviates cognitive arousal prior to sleep. This reduction in arousal can enhance sleep continuity by decreasing sleep onset latency. On the other hand, activity regulation implemented through behavioral activation techniques may disrupt the overrest-overactivity cycle commonly observed in FMS, thereby restoring both physical functionality and sleep pressure. Furthermore, gradual exposure techniques reduce movement-related anxiety and increase patients’ daytime physical activity, which positively influences sleep quality. Collectively, these mechanisms may contribute to reduced disease severity. The noted decrease in the FIQ score within the intervention group indicates a clinically significant reduction in total disease burden, encompassing physical function, pain intensity, exhaustion, and emotional symptoms. This extent of change is anticipated to yield quantifiable improvements in patients’ everyday activities and quality of life, underscoring the practical importance of personalized CBT beyond its statistical significance.

A significant reduction in BHS scores was observed in the intervention group following CBT. Hopelessness is a key component of cognitive predisposition to pain because it reflects pessimistic future expectations and negatively impacts chronic pain patients’ functional results [31]. However, research systematically monitoring this construct with independent measurement tools within CBT interventions remains limited. Chronic pain progressively diminishes patients’ belief in recovery and contributes to the formation of a pessimistic cognitive schema about the future. This cycle impedes treatment adherence and exacerbates the psychosocial burden of the disease [7]. The results indicate that individual CBT not only alleviates existing symptoms but also modifies negative cognitive schemas. These findings suggest that hopelessness should be addressed as an independent therapeutic target in FMS management.

Several limitations should be acknowledged. The relatively small sample size restricts the generalizability of the findings. A formal a priori sample size calculation was not conducted, thereby limiting the statistical power of the investigation; this should be taken into account when interpreting the results. As the study was conducted at a single tertiary care center, caution is warranted when applying these results to other healthcare settings. Participant blinding was not feasible due to the group assignment process, which increases the risk of response bias. A further limitation is the lack of long-term follow-up data, which prevents assessment of the durability of treatment effects beyond the intervention period. Additionally, all outcome measures relied solely on self-report scales, making them vulnerable to response bias and social desirability effects and potentially limiting their ability to capture objective functional or physiological changes. The high drop rate observed in the intervention group may have reduced the statistical power. The lack of an intention-to-treat analysis constitutes an additional limitation. Because post-treatment data were unavailable for participants who discontinued, imputation was not possible. Consequently, analyses were limited to completers, which may have introduced selection bias and should be taken into account when interpreting the results. Moreover, the per-protocol approach is likely to overestimate treatment effects relative to those observed in real-world clinical settings. Nonparametric tests are appropriate for the current sample size and data distribution, given the covariate, and for controlling for baseline differences. Future studies with larger samples should consider ANCOVA or mixed-effects models to obtain more robust estimates of treatment effects.

## Conclusion

This randomized controlled trial demonstrates that individualized CBT, when added to routine treatment, yields significant improvements in anxiety, depression, sleep quality, disease severity, and hopelessness among patients with FMS. These findings contribute to the growing body of evidence supporting CBT as an integral component of holistic FMS management. The integration of individualized CBT protocols into clinical practice is suggested as part of multidisciplinary approaches to FMS care. Further research involving larger sample sizes, extended follow-up periods, and diverse patient subgroups is necessary to strengthen the evidence base in this field.

## Supplementary Information

Below is the link to the electronic supplementary material.

Supplementary Material 1

## Declarations

### Open data sharing note

Raw data can be shared if requested.
