# Supplementary material for: Individualized cognitive-behavioral therapy improves anxiety, depression, sleep quality, hopelessness, and disease severity in fibromyalgia syndrome: a single-blind randomized controlled trial
Source: Rheumatol Int. 2026 May 26;46(6):114. doi: 10.1007/s00296-026-06146-9 (PMC13212815; doi:10.1007/s00296-026-06146-9)
Supplement: Supplementary file 1 — Supplementary Material 1 [file 296_2026_6146_MOESM1_ESM.docx]

**CONSORT 2010 Checklist**

*Checklist of information to include when reporting a randomised trial*

Manuscript: The Effectiveness of Individualized Cognitive-Behavioral Therapy in Fibromyalgia Syndrome: A Randomized Controlled Trial

| **Section / Topic** | **Item No** | **Checklist Item** | **Reported on Page / Location** |
| --- | --- | --- | --- |
| Title and abstract | 1a | Identification as a randomised trial in the title | Title: '…A Randomized Controlled Trial' |
|  | 1b | Structured summary of trial design, methods, results, and conclusions | Abstract (Introduction, Methods, Results, Conclusion) |
| Introduction – Background and objectives | 2a | Scientific background and explanation of rationale | Introduction (paragraphs 1–3) |
|  | 2b | Specific objectives or hypotheses | Introduction (final paragraph) |
| Methods – Trial design | 3a | Description of trial design (parallel) including allocation ratio (1:1) | Methods – first paragraph; single-blind parallel RCT, 1:1 ratio |
|  | 3b | Important changes to methods after trial commencement, with reasons | No changes were made after trial commencement – Not applicable |
| Methods – Participants | 4a | Eligibility criteria for participants | Methods – inclusion and exclusion criteria described |
|  | 4b | Settings and locations where the data were collected | Methods – tertiary Physical Medicine and Rehabilitation clinic |
| Methods – Interventions | 5 | Interventions for each group with sufficient details to allow replication | Methods – CBT section (10 weekly individual sessions, 50–60 min; techniques described) |
| Methods – Outcomes | 6a | Completely defined pre-specified primary and secondary outcome measures | Methods – Data assessment section (HADS, FIQ, JSS, BHS) |
|  | 6b | Any changes to trial outcomes after the trial commenced, with reasons | No changes were made to outcomes – Not applicable |
| Methods – Sample size | 7a | How sample size was determined | Not explicitly reported – acknowledged as a limitation |
|  | 7b | When applicable, explanation of any interim analyses and stopping guidelines | No interim analyses were conducted – Not applicable |
| Methods – Randomisation – Sequence generation | 8a | Method used to generate the random allocation sequence | Methods – Randomization procedure (sealed opaque envelopes, 1:1 ratio) |
|  | 8b | Type of randomisation; details of any restriction | Simple randomisation; no further restriction specified |
| Methods – Allocation concealment mechanism | 9 | Mechanism used to implement the random allocation sequence | Methods – sealed opaque envelopes |
| Methods – Implementation | 10 | Who generated the allocation sequence, who enrolled participants, and who assigned participants to interventions | Methods – single clinical psychologist administered CBT; physician conducted assessments |
| Methods – Blinding | 11a | If done, who was blinded after assignment to interventions | Methods – single-blind; outcome assessor (physician) blinded to group assignment |
|  | 11b | If relevant, description of the similarity of interventions | Not applicable (non-pharmacological intervention) |
| Methods – Statistical methods | 12a | Statistical methods used to compare groups for primary and secondary outcomes | Methods – Shapiro-Wilk, Mann-Whitney U, Wilcoxon signed-rank, Chi-square; IBM SPSS v20 |
|  | 12b | Methods for additional analyses, such as subgroup analyses and adjusted analyses | Methods – Delta (change) scores calculated and compared between groups (Mann-Whitney U) |
| Results – Participant flow | 13a | For each group, the numbers of participants who were randomly assigned, received intended treatment, and were analysed for the primary outcome | Results – paragraph 1; Figure 1 (CONSORT flow diagram) |
|  | 13b | For each group, losses and exclusions after randomisation, with reasons | Results – paragraph 1 (reasons: irregular attendance, pharmacological changes, surgery, loss to follow-up) |
| Results – Recruitment | 14a | Dates defining the periods of recruitment and follow-up | Methods – July 15, 2025 to January 15, 2026 |
|  | 14b | Why the trial ended or was stopped | Trial completed as planned; no early stopping |
| Results – Baseline data | 15 | A table showing baseline demographic and clinical characteristics for each group | Results – Table 1 |
| Results – Numbers analysed | 16 | For each group, number of participants included in each analysis | Results – Intervention group: n=23; Control group: n=34 |
| Results – Outcomes and estimation | 17a | For each primary and secondary outcome, results for each group, effect size and its precision | Results – Table 2 (median scores, p-values, delta scores) |
|  | 17b | For binary outcomes, presentation of both absolute and relative effect sizes | Not applicable – no binary outcomes |
| Results – Ancillary analyses | 18 | Results of any other analyses performed, including subgroup analyses and adjusted analyses | Results – between-group delta score comparisons (Table 2) |
| Results – Harms | 19 | All important harms or unintended effects in each group | Not reported – acknowledged as a limitation |
| Discussion – Limitations | 20 | Trial limitations, addressing sources of potential bias, imprecision, and multiplicity of analyses | Discussion – Limitations paragraph (sample size, single center, self-report scales, no follow-up, drop-out, no trial registration) |
| Discussion – Generalisability | 21 | Generalisability (external validity, applicability) of the trial findings | Discussion – Limitations paragraph (single tertiary care center) |
| Discussion – Interpretation | 22 | Interpretation consistent with results, balancing benefits and harms, and considering other relevant evidence | Discussion and Conclusion sections |
| Other information – Registration | 23 | Registration number and name of trial registry | This trial was not prospectively registered in a clinical trial registry |
| Other information – Protocol | 24 | Where the full trial protocol can be accessed, if available | Protocol not publicly available |
| Other information – Funding | 25 | Sources of funding and other support; role of funders | Funding section – TÜBİTAK open-access support to be applied after acceptance |

** We strongly recommend reading this checklist in conjunction with the CONSORT 2010 Explanation and Elaboration.*

*Reference: Schulz KF, Altman DG, Moher D, for the CONSORT Group. CONSORT 2010 Statement: updated guidelines for reporting parallel group randomised trials. BMJ 2010; 340:c332.*
